# Improved Physicochemical and Structural Properties of Blueberries by High Hydrostatic Pressure Processing

**DOI:** 10.3390/foods8070272

**Published:** 2019-07-21

**Authors:** Maria Paciulli, Ilce Gabriela Medina Meza, Massimiliano Rinaldi, Tommaso Ganino, Alessandro Pugliese, Margherita Rodolfi, Davide Barbanti, Michele Morbarigazzi, Emma Chiavaro

**Affiliations:** 1 Department of Food and Drug, University of Parma, Parco Area delle Scienze 27/A, 43124 Parma, Italy; 2 Department of Biosystems and Agricultural Engineering, Michigan State University, East Lansing, MI 48824-1323, USA; 3 Consiglio Nazionale delle Ricerche, Istituto per la Valorizzazione del Legno e delle Specie Arboree (IVaLSA), 50019 Sesto Fiorentino, Italy; 4 HPP Italia, Via E. Carbognani 6, Traversetolo, 43029 PR, Italy

**Keywords:** high pressure, blanching, fruit, microscopy, pectin methyl esterase, texture, color, antioxidant activity

## Abstract

The use of high pressure on fruits and vegetables is today widely studied as an alternative to the traditional thermal preservation techniques, with the aim of better preserving nutritional and organoleptic properties. The use of high hydrostatic pressures (400–600 MPa; 1–5 min; room temperature) was tested on the physicochemical and structural properties of blueberries, in comparison to raw and blanched samples. High hydrostatic pressures led to higher tissue damages than blanching, related to the intensity of the treatment. The cellular damages resulted in leakage of intracellular components, such as bioactive molecules and enzymes. As a consequence, among the high pressure treatments, the resulting antioxidant activity was higher for samples treated for longer times (5 min). Pectinmethyl esterase (PME), deactivated by blanching, but strongly barotolerant, was more active in blueberries treated with the more intense high pressure conditions. Blueberry texture was better retained after high pressure than blanching, probably because of the PME effect. Blueberry color shifted towards purple tones after all of the treatments, which was more affected by blanching. Principal component analysis revealed the mild impact of high pressure treatments on the organoleptic properties of blueberries.

## 1. Introduction

Blueberries are nowadays a very popular fruit because of their sensory and health related properties. Blueberries, indeed, in addition to being appreciated for their color, flavor, texture, and juiciness, are also a natural source of bioactive compounds. The so-called polyphenols, including anthocyanins, the molecules responsible for blueberry color, are powerful antioxidants with recognized anti-inflammatory and antihypertensive properties, in addition to being involved in many cell regulation pathways [1]. 

Blueberries are often consumed fresh, but untreated fruit show short storage life, usually due to both microbial and enzymatic spoilage. The presence of enzymes such as peroxidase (POD), polyphenol oxidase (PPO), lipoxygenase (LOX), and lipase may be responsible for color and flavor changes, while pectin-methylesterase (PME) and polygalacturonase (PG) are involved in texture modifications [2]. The application of preservation processes, in order to guarantee consumer safety and extend shelf life, thus, became necessary. Blueberries are generally sold frozen, dehydrated, or heat treated. Such treatments induce important modifications in the product quality. Traditional thermal preservation methods, such as canning, make them susceptible to significant losses in their natural sensorial (i.e., aroma, flavor) and nutritional (bioactive compounds) properties [3]. Moreover, freezing and drying have a huge effect on fruit structure and texture [4]. 

Retention of nutritional value and freshness of fruit and vegetables (F&V) is a major challenge for the food industry. Novel processing technologies can address these issues. Among them, high hydrostatic pressure (HHP) is an attractive technology, because of the use of low temperatures during processing (i.e., room temperature), combined with high hydrostatic pressures (100–1000 MPa) and short time (a few seconds or minutes). HHP belongs to the so-called “non-thermal technologies”, which are capable of retaining low molecular weight food compounds (i.e., flavoring agents, pigments, and vitamins) by not affecting covalent bonds [5,6]. Additionally, recent attention has been paid to the effects of HHP on the color attributes of F&V, since HHP can increase the intensity of color characteristics because of cell lysis and subsequent leakage of pigments [7]. The effect of high hydrostatic pressure (HHP) on the improvement of the technological functionalities of polymers, such as proteins and polysaccharides, has been recently studied [8,9]. Texture is an important quality attribute in F&V, and indicates freshness from a consumer point-of-view. The structural integrity and texture of F&V is attributed mainly to the primary cell wall, the middle lamella, and the turgor generated within cells by osmosis [10]. The primary cell wall basic structure consists of a cellulose–hemicellulose network [11]. The firmness developed by these two polysaccharides is not affected by processing or storage; however, pectin is affected by both enzymatic and non-enzymatic reactions. Pectin is the main constituent of the middle lamella, and gives firmness and elasticity to tissues [12]. All of these positive results indicate that HHP may be useful for retaining major structural quality characteristics of F&V.

Based on our knowledge, the application of HHP on whole blueberries has mainly been used to investigate microbial inactivation [13] or biomolecule extraction [14]. No studies are available on the effect of high hydrostatic pressure on the physicochemical and structural properties of whole blueberry fruit. The aim of this study was to evaluate the related properties affecting blueberry structure, color quality, and antioxidant capacity after HHP treatments in comparison with conventional thermal processing.

## 2. Material and Methods

### 2.1. Sample Preparation

Blueberries (*Vaccinium corymbosum*, cv. Duke) were purchased from a local market (Parma, Italy). After washing and draining, whole samples from the same batch and with similar dimensions (10 ± 0.1 mm) were selected and used for the study. 

### 2.2. Treatments

For each sample, six conditions were analyzed: Raw (R), Blanched (BL), and treated with high hydrostatic pressure under four different conditions, as described later.

#### 2.2.1. Blanching 

Blueberries were blanched in a Combi-Steam SL (V-Zug, Zurich, Switzerland) oven, which presented an internal volume of 0.032 m^3^, an air speed of 0.5 m s^−1^, and a steam injection rate of 0.03 kg min^−1^, at 95 °C for two min in accordance with Sablani et al. [15]. Nine samples were arranged in a circle and one was placed at the center to ensure uniform heating conditions. The treatment was conducted in triplicate. 

#### 2.2.2. High Hydrostatic Pressure 

The treatments were conducted in a 300 L high pressure plant (Avure Technologies Inc., Kent, WA, USA), at the “HPP Italia” of Traversetolo (Italy). Ten blueberries were vacuum sealed inside flexible (75 mm thickness) plastic pouches (Ultravac Solutions, Kansas City, MO, USA). Cold water (4 °C) was used as pressure medium. HHP treatments were conducted at 400 and 600 MPa, both for 1 and 5 min; the samples were, thus, called 400-1, 400-5, 600-1, and 600-5. These conditions were chosen on the basis of preliminary experiments performed on the same types of samples [16]. Treatments were conducted at room temperature (20 ± 1 °C). Temperature increase due to compression was not higher than 2–3 °C/100 MPa. Three pouches were processed and analyzed for each treatment condition.

### 2.3. Moisture Content 

Moisture content was determined according to the Association of Official Agricultural Chemists (AOAC) method [17] on both raw and treated samples. Almost 5 g of homogenized sample (as triplicate) were dried in a convection oven (ISCO NSV 9035, ISCO, Milan, Italy) at 105 °C for at least 16 h until constant weight was reached. The samples were stored at 4 °C and their moisture content was determined one day after HHP and thermal treatments. 

### 2.4. Histological Analysis

The samples were fixed in formalin, acetic acid, alcohol (FAA) solution (formalin: acetic acid: 60% ethanol solution, 2:1:17 *v*/*v*) [18]. After two weeks, they were dehydrated using increasingly concentrated alcoholic solutions. The inclusion was made in a methacrylate resin (Technovit 7100, Heraeus Kulzer and Co., Wehrheim, Germany) and the resulting blocks were sectioned with transversal cuts at 3 μm thickness using a semithin Leitz 1512 microtome (Leitz, Wetzlar, Germany). The sections were stained with Toluidine Blue (TBO) and with a solution containing FeSO4 for the tannin analysis [18]. Four pieces of fruit were sampled for each treatment. Sections were observed with a Leica DM 4000B optical microscope (Leica Imaging Systems Ltd., Wetzlar, Germany) equipped with a Leica DMC 2900 digital camera (Leica Imaging Systems Ltd., Wetzlar, Germany). The tissues were measured using an image analysis system (LAS v4.10.0, Leica Application Suite, Wetzlar, Germany). The microscopic observations were carried out by observing at least ten slides carrying ten sections each, for each specimen. The image analyses were carried out using a manual configuration of the image analysis system. 

### 2.5. DPPH Free Radical Scavenging Activity Test

Antioxidant molecules were extracted from 1 g of ground sample, using 5 mL of a methanol/water (70:30 *v*/*v*) solution, kept under motion for 60 min at room temperature, and then paper filtered. The solvent was evaporated, and the extract was then dissolved in 10 mL of methanol, thus centrifuged at 5040× g for 15 min at 4 °C. Analyses were performed in triplicate mixing 100 μL of surnatant and 1 mL of 2,2-Diphenyl-1-picrylhydrazyl (DPPH) methanolic solution (0.2 mm), bringing the mix to a final volume of 2.4 mL with methanol. The absorbance of the solution was recorded at 517 nm by a Perkin Elmer UV-Visible spectrophotometer after an incubation time of 30 min in the dark at room temperature. Blank was prepared and analyzed following the same procedure, using 100 μL of methanol instead of sample. The radical scavenging activity was calculated as follows:

I% = ((Abs0 − Abs1)/Abs0) × 100,(1)

where Abs0 was the absorbance of the blank and Abs1 was the absorbance of the sample. The Trolox Equivalent Antioxidant Capacity value (TEAC) expressed as μmol Trolox equivalents/gram of dry weight (μmol TE/g of dw) of the samples was calculated from the calibration curve obtained by measuring the absorbance at 517 nm of Trolox methanolic solutions at different concentrations. Three replicates were analyzed for each treatment condition. 

### 2.6. Pectin Methylesterase (PME) Activity

The enzyme extraction and the determination of its activity were conducted according to the method of Vicente et al. [19]. Five grams of sample were ground with 15 mL of 1 M NaCl (1:3, *w*/*v*) containing 1% (*w*/*v*) of polyvinylpolypyrrolidone (PVPP). The suspension obtained was stirred for 4 h and then centrifuged at 10,000× *g* for 30 min. The supernatant was collected, adjusted to pH 7.5 with 1 N NaOH, and used for assaying the enzyme activity. The activity was assayed in a mixture containing 600 μL of 0.5% (*w*/*v*) pectin, 150 μL of 0.01% bromothymol blue pH 7.5, 100 μL of water pH 7.5, and 150 μL of enzymatic extract. PME activity results in a progressive discoloration of the blue solution. The reduction of the absorbance at 620 nm was measured every 15 s for two minutes. The PME activity was calculated using the slope of a linear segment absorbance-time [20]. Percentage variations were calculated in comparison to the raw sample. Three replicates were analyzed for each treatment condition.

### 2.7. Texture Analysis

The texture of raw and treated samples was analyzed using a TA.XT2i Texture Analyzer equipped with a 25 kg load cell (Stable Micro Systems, Godalming, UK), a force resolution equal to 0.01 N, and an accuracy value of 0.025%. Puncture test (trigger force 0.05 N) was performed using a 2 mm diameter stainless steel needle probe, driven up in a radial direction to the center of the samples at a speed of 1 mm s^−1^, following the method proposed by Paciulli et al. [21]. 

The following parameters were determined from the force vs. time curves: first peak force (F_P1_ given in N), which indicates the resistance opposed by external cell layers to needle penetration [21]; maximum puncture force (F_max_ given in N), which indicates the resistance opposed by the pulp to needle penetration; and Area under the force/time curve (Area given in N*s), which represents the total work carried out by the needle probe to penetrate the sample. The parameters were quantified using the application software provided (Texture Exponent for Windows, version 6.1 10.0). Ten blueberries units were analyzed for each treatment.

### 2.8. Color

Color determination was carried out using a Minolta Colorimeter (CM2600d, Minolta Co., Osaka, Japan) equipped with a standard illuminant D65, which simulates natural noon daylight in order to mimic the vision of the human eye. The measurement was performed on two opposite points on the blueberries epidermis. The instrument was calibrated before each analysis with white and black standard tiles. L* (lightness; black = 0,white = 100), a* (redness > 0, greenness < 0), b* (yellowness > 0, blue < 0), C (chroma, 0 at the centre of the color sphere), and h° (hue angle, red = 0°, yellow = 90°, green = 180°, blue = 270°) were quantified on each fruit using a 10° position of the standard observer (Commission Internationale de l’eclairage), [22]). The ΔE for all the treated samples in comparison to the raw vegetables was also calculated. 10 determinations were performed for each treatment. 

### 2.9. Statistical Analysis

One-way analysis of variance (ANOVA) among all the different treated samples and two way-ANOVA among the HHP treated samples, using pressure and time as independent variables, were performed using Statistical Package for Social Science (SPSS) software (Version 25.0, SPSS Inc., Chicago, IL, USA). A Least Significant Difference (LSD) post-hoc test at a 95% confidence level (*p* ≤ 0.05) was performed to further identify differences among treatments. 

Pearson correlation coefficients were calculated among all variables considering 95% and 99% confidence levels (*p* < 0.05 and *p* < 0.01).

Principal component analysis (PCA) was performed using the normalized variables, as reported by Medina Meza et al. [23]. Before running PCA, factor analysis (FA) was applied to exclude the variables that showed low contribution to explain the variance. FA was carried out on 10 independent variables and only parameters with factor loadings higher than 0.70 were used for PCA, plotting them versus all cases (samples).

## 3. Results and Discussion

### 3.1. Histological Analysis

**Figure 1:**
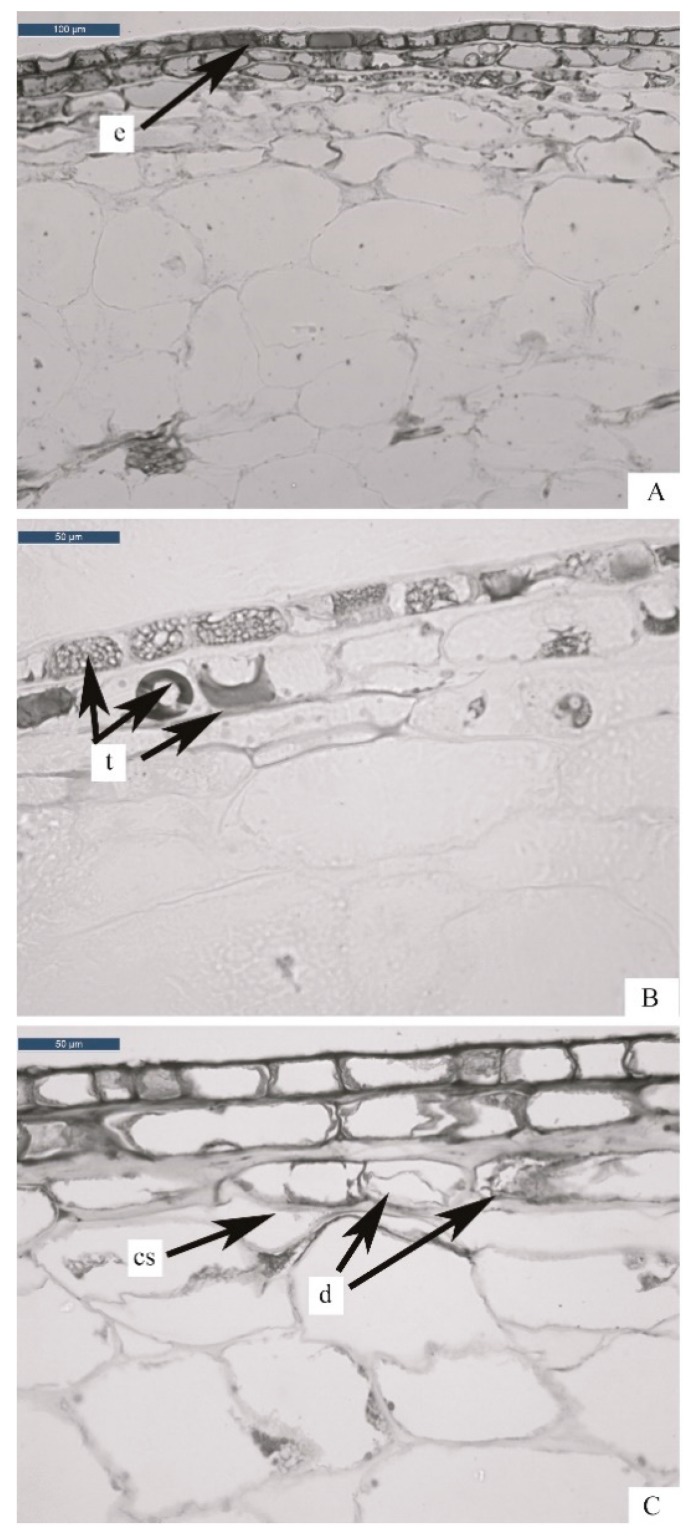
Transverse sections of blueberries samples: (**A**) raw or uncooked stained with Toluidine Blue (TBO); (**B**) raw or uncooked stained with tannin solution; (**C**) blanched stained with Toluidine Blue (TBO). Legend: e = epidermis; t = tannins; cs = cell separation; d = dehydration.

Raw blueberries fruits showed an epidermis composed of a single layer of cells (Figure 1A), with abundant tannin inclusions (Figure 1B). The subdermis, located immediately under the skin, was composed of 2 or 3 layers of cells, and showed multiple solid tannin inclusions (Figure 1B). The epidermis and subdermis exhibited thickened cell walls. These layers contain the pigments. Mesocarp, composed of parenchymatic cells, showed thin layers and large vacuoles (Figure 1A). From the observation of the transversal section stained with Tannin Solution, the presence of tannins was perceived mainly in the epidermis and subdermis (Figure 1B). In these cellular layers, tannins were solid and crystalline, while in the mesocarp parenchymatic tissue, tannins appeared as single inclusions or “crystalline powder”. Zifkin and collaborators [24], in a study on blueberries, revealed that flavonols (condensed tannins) are among the major antioxidant compounds in epidermis and subdermis.

After blanching, blueberry microstructure did not show clear alterations in comparison to raw samples. Subdermis cells showed swollen cell walls due to the absorption of intra or extracellular water; this effect is called gelatinization. The major variations of blueberry tissues after blanching treatment were: cellular dehydration, cell wall gelatinization, and cell separation. Mesocarp cells, after thermal treatment, showed dehydration symptoms, with the detachment of the cellular membrane from the wall. In Figure 1C, it is also possible to observe cell separation with formation of large intracellular spaces between parenchymatic cells. The same observations were previously reported by Fuchigami et al. [25] in a study on carrots subjected to slight cooking, and by Paciulli and collaborators [21] in a study on blanched asparagus. As confirmed by several authors [21,26,27,28], cell separation is due to the breakage of chemical bonds between the pectic components of the middle lamellae of adjacent cells or to hydrolysis of some other components of the cell wall, such as pectin, hemicellulose, and cellulose. Compared to raw samples, tannins showed shape mild alteration and apparently a higher concentration. These compounds were visible in the epidermis, subdermis, mesocarp, and near the seeds. The blanching treatment seemed to induce leaking of tannins from cellular walls. Zaupa and coworkers [29] observed the same effect in different rice cultivars, where total antioxidant activity of samples increased after thermal treatments. 

**Figure 2:**
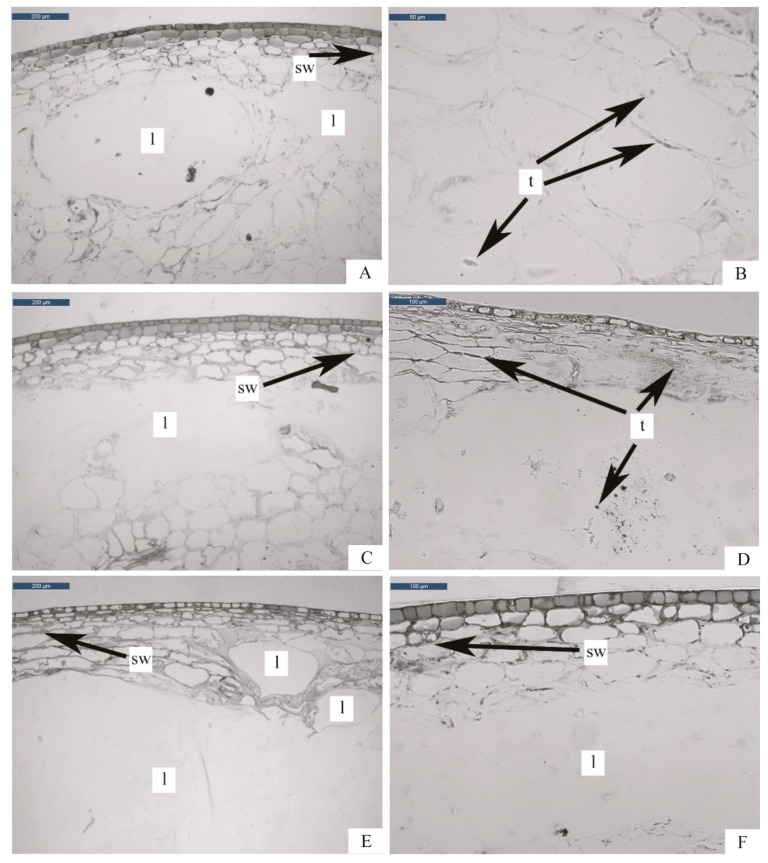
Transverse sections of blueberries samples treated using HHP technology: (**A**) 400-1 stained with Toluidine Blue (TBO); (**B**) 400-1 stained with tannin solution; (**C**) 400-5 stained with Toluidine Blue (TBO); (**D**) 400-5 stained with tannin solution; (**E**) 600-1 stained with Toluidine Blue (TBO); (**F**) 600-5 stained with Toluidine Blue (TBO). Abbreviations: l = lacuna; sw = swelling; t = tannins.

The high pressure treatment at 400 MPa for 1 min (400-1) induced separation of the external cells layers (epidermis and few layers of mesocarp). Cells seemed to be deformed by the appearance of air bubbles, and as a consequence, elliptical or circular lacunas are shown (Figure 2A). The same effects were previously observed by Prestamo and Arroyo [30] in a study on spinach and broccoli exposed to high pressures. Similarly to our observations, Tangwongchai et al. [31] observed the formation of large cavities in tissues of cherry tomatoes treated by high pressures of between 200 and 400 MPa. These authors hypothesized that during depressurization, the previously compressed air expanded rapidly, aggregating into larger bubbles, which caused the formation of cavities. Moreover, in our experimental conditions, mesocarp cells appeared dehydrated with gelatinized walls; in some cases, cells separation and cell wall breakage are also shown (Figure 2A). 

The use of Tannin Solution staining on blueberry sections highlighted the presence of high quantities of tannins mainly in the epidermis and mesocarp (Figure 2B). The literature does not explain the increase of tannins in blueberries after high pressure treatments, but it is clear that the exposition of berries to high pressures increases the extraction of these antioxidant compounds. Serment-Moreno and collaborators [10], in their review, observed that the major role in the variation of polyphenol content after 200–600 MPa treatment is their release after disruption of cellular membranes and possible degradation due to their high susceptibility to oxidation and enzymatic reactions, however, details of the specific reaction taking place still remain unknown.

In Figure 2C the microstructure of blueberries treated at 400 MPa for 5 min (400-5) is shown. The tissue appeared disorganized and composed by shapeless cells that have lost their turgidity and, in comparison to the shorter treatment (400-1), more intercellular spaces were observed. Mesocarp resulted collapsed with formation of circular and elliptic cavities from the subdermis to the inner parenchyma (Figure 2C), probably due to the effect of the air bubbles aggregation during depressurization [31]. Cavities resulted larger if compared to the ones found in 400-1 (Figure 2A). Moreover, epidermis, subdermis and the firsts layer of mesocarp resulted detached; cell walls appeared thickened (gelatinized) all over the microstructure and the entire tissue showed considerable damages. Tannins solution stain highlights the presence of large amount of tannins that cover the entire structure (Figure 2D). 

Blueberries treated at 600 MPa for 1 min (600-1) reflected the observations done for 400-1. The degree of parenchyma gelatinization, the dehydration of the external mesocarp tissues, as well as the presence of tannins, resulted comparable for 400-1 and 600-1(Figure 2E). 

After the treatment at 600 MPa for 5 min, blueberry tissues revealed evident damages, especially near the epidermis, where deep cavities, dehydration and gelatinization are present (Figure 2F). This treatment showed effects on the final product similar to those observed for HHP 400-5. Also tannins, as seen for 400-5, resulted distributed in all the structure. 

Blanching and high pressures caused changes in the cellular structure of blueberries under the effect of different phenomena: chemical and mechanical, respectively. Indeed, while blanching mainly led to hydrolysis of pectin chemical bonds, causing cell separation, high pressures brought to localized air bubbles explosions, breaking groups of cells. Blanching appeared thus less invasive than high pressure, although it involves widespread changes in the entire structure. On the other hand high pressures treatments, despite their strength, provoked localized damages. The extent of the cellular damages due to high pressures resulted more influenced by treatment time than pressure intensity. 

### 3.2. Antioxidant Activity (DPPH)

In Table 1 are reported the values of blueberries antioxidant activity measured by DPPH assay. According to Zifkin et al. [24], the molecules responsible for antioxidant activity in blueberries are flavonols (condensed tannins), proantocyanidins and anthocyanins in epidermis and subdermis, anthocyanins in mesocarp, flavanols in placenta. These molecules act as radical scavengers [32].

Raw samples showed values of about 105 μmolTE/g_dw_; this value is in line with the results of Lohachoompol et al. [32], with slight differences attributable to the different blueberries varieties. 

All the treatments affected blueberries antioxidant activity in comparison to raw samples. Blanching decreased it slightly, despite tannins, resulted better extracted from the cell walls (Par.3.1). Brownmiller et al. [3] registered a decrease of the total antioxidant activity after blueberries blanching, despite they didn’t observe any anthocyanins reduction, probably because of other antioxidant molecules losses. Conversely, Rossi et al. [33] observed higher antioxidant activity in blueberries juice after fruits blanching, probably because of the rapid polyphenoloxidase inactivation and/or increase in extraction yield, due to heat induced skin permeability [34]. 

The high pressure treated samples showed behaviors similar to the blanched ones (Table 1). On the other hand, in comparison to R, 400-1 and 600-1 showed a significantly lower antioxidant activity, with losses of around 34 and 27%, respectively. 600-5 resulted instead the less impacting treatment with around 82% retention of the total activity. Two-way ANOVA, conducted among the HHP treated samples, revealed a time-dependent behavior: the longer the time, the higher the antioxidant activity. The retention of the antioxidant activity with the increase of the pressure holding time may be explained as a better extraction of the bioactive molecules from the broken cells, as suggested by other authors [20] and confirmed by the histological observations (Figure 2). Moreover it’s known that the total antioxidant activity in blueberries is due to several classes of molecules, differently distributed among the tissues [24], that can thus be extracted under different conditions. The highest loss of antioxidant activity registered for 400-1 and 600-1 may be also attributable to the poor inactivation of PPO and POD, the main enzymes responsible for phenol decay [35].

### 3.3. Pectin Methylesterase (PME) Activity 

Pectin methyl esterase (PME) is the enzyme responsible for the demethylesterification of plant cell walls pectin. This enzyme, in combination with polygalacturonase, affects texture of fruit and vegetables during postharvest storage [35]. 

In Table 1, the blueberry PME activity is reported as percentage variation in comparison to the raw sample. Among treatments, blanching, with a residual activity of 10%, was the most effective on PME inactivation, because of the protein thermal denaturation [36]. On the other hand, high pressure treatments had lower effect on PME inactivation. HHP400-1, leading to a residual PME activity of around 65%, resulted the most effective high-pressure treatment. The high PME baro-resistance is reported for many fruit and vegetables [37]. It has been shown that, among many studied products, tomato PME is the most pressure-resistant, even being inactivated at ambient conditions up to 800 MPa [37]. The two-way ANOVA, performed among the four HHP treatments, revealed a time dependence for blueberry PME inactivation by high pressure—the higher the pressure holding time, the lower the inactivation. Similarly, Paciulli et al. [20] reported increased enzymatic activity for longer pressure exposure times on beetroot slices. These authors explained this phenomenon with enzymes leaking from the broken cells, which leading to easier contact with the substrates, results in higher activities. This hypothesis follows the same trend of the antioxidant activity and is supported by the histological observations that show 400-5 and 600-5 as the most damaged tissues (Figure 2C–F). Phenomena of enzyme activation, under the effect of high pressure, due to conformational changes have also been reported [38]. A significant interaction of time and pressure was also revealed by the two-way ANOVA; indeed, 400-5 showed almost no effect on PME. In contrast, in a study on two different varieties of pumpkin, Paciulli et al. [39] reported high-pressure treatment at 400 MPa for 5 min as the most effective against PME, with a residual activity of 20–25%; this demonstrates that the PME barotolerance is specie dependent. 

### 3.4. Texture

**Figure 3:**
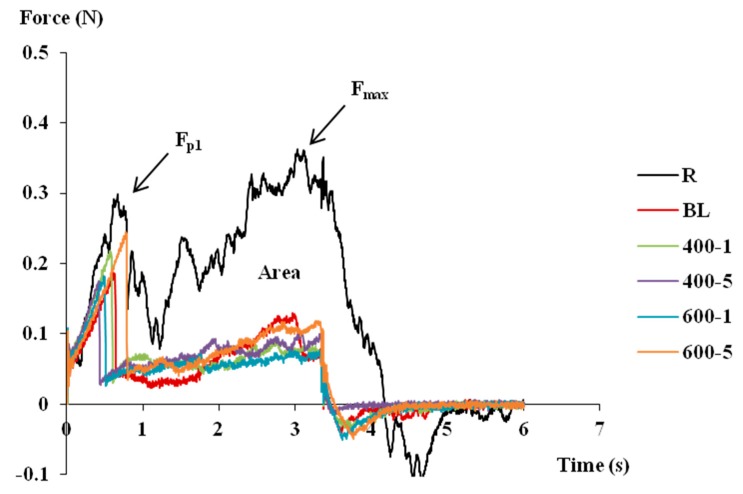
Texture analysis profiles of raw and treated blueberries obtained by puncture test. Abbreviations: R = raw or untreated; BL = blanched; 400-1 = HHP at 400 MPa for 1 min; 400-5 = HHP at 400 MPa for 5 min; 600-1 = HHP at 600 MPa for 1 min; 600-5 = HHP at 600 MPa for 5 min; F_P1_ = maximum force first peak; F_max_ = absolute maximum force; Area = area under the force/time curve.

Figure 3 reports the texture analysis profiles of blueberries in all the studied conditions, obtained by puncture test. In the same figure, the parameters F_p1_, F_max_, and Area are shown, and their values summarized in Table 2. The absence of significant differences in water content (Table 1) between raw and treated samples indicates that the observed texture differences were not related to the cellular turgidity, but they strictly depend on the tissues structure, as modified by the treatments. 

The first peak force (Fp_1_) was generated in blueberries by epidermal and subdermal cells, characterized by cutinization and thickened cell walls (Figure 1A, Section 3.1). It is visible how untreated blueberries showed the highest FP_1_ (~0.30 N) among all samples. Blanching led to the highest FP_1_ drop among all treatments. This phenomenon, already reported for other blanched vegetables [21], may be ascribable to the external cell wall swelling (Figure 1C, Par.3.1) and pectin thermal degradation, with consequent softening. HHP treated samples showed Fp_1_ values significantly lower than R, with the exception of 600-5, which showed values of Fp_1_ around 0.25 N, slightly higher than BL. Fp_1_ reduction under high pressure can be justified by the detachment of the epidermis, subdermis, and first mesocarp layers, as well as cell wall swelling, as observed from the histological analysis (Figure 2). The two-way ANOVA performed among the high-pressure treated samples (Table 1) showed a synergistic effect of pressure and time on Fp_1_. Texture recovery during pressure holding time was already reported by other authors [20,40] and associated to the insufficient inactivation of PME, whose reaction product is the low-methoxy pectin, that forms a gel-networkwith divalent ions such as Ca and Mg, contributing to the enhanced hardness value. This hypothesis, more evident at 600 MPa, is supported by the PME activity results (Table 1). At 400 MPa, the effect of the tissue damage prevails. 

Comparing F_max_ with Fp_1_ (Table 2), it is visible how, after treatments, Fp_1_ was almost 50% higher than F_max_, indicating that the skin had the major effect on blueberries mechanical properties. Moreover, F_max_ of all the treated samples was significantly lower than the raw ones (Table 2); this phenomenon may be justified by an easier penetration of the needle probe across the dehydrated and gelled parenchyma of the blanched (Figure 1C, Section 3.1) and HHP treated blueberries (Figure 2, Par.3.1). Among the HHP treated samples, 600-5 was significantly firmer than the others. Texture recovery after prolonged exposures to high pressure has already been reported [40]. This phenomenon, as discussed for Fp_1_, can be justified with the high PME activity. 

Area values comprise both the effect of skin and pulp penetration. It has been confirmed that all of the treatments were detrimental for blueberry texture, showing significantly lower Area values in comparison to the untreated samples. Despite BL samples showing the lowest values among all samples (~0.22 N*s), only 600-5 (~0.36 N*s) was significantly higher than BL. The two-way ANOVA confirmed a time dependence of the Area values under the effect of pressure—the higher the exposure time, the higher the Area values. This phenomenon, already reported in previous studies, was justified either with tissue recovery during the holding time [41], or with the still high PME activity [42]. Confirming the last hypothesis, a positive correlation was found between Area values and PME (*R* = 0.498; *p* < 0.05); with increasing PME activity, the firmness of blueberries increased. 

### 3.5. Color

The bluish blueberry skin color is affected by anthocyanin content, as well as by the presence of surface waxes [43]. L*, a*, and b* values, measured on raw samples (Table 3), are in line with data already reported for different varieties of blueberries [43]. After blanching, blueberries were slightly darker than the raw samples, with lower L* values (Table 3); this may be related to melting of blueberries’ cutaneous wax [44]. At the same time, a* and b* increase, indicating enhancement of the perceived red and blue colors. The slight increase of the color intensity C may be instead ascribed to alterations of the surface reflecting properties. Similarly, Mazzeo et al. [45] found an enhancement of the asparagus, green bean, and zucchini green color after blanching; they associated it to a change of the color perception due to the air replacement with water and cell juices. The shift of h° from about 270 to 350 degrees confirms the shift to a red-purple color. According to ΔE values, blanching was the more affecting treatment. According to previous studies, thermal treatments are more detrimental than high pressure on the anthocyanin content of fruits, with consequent higher color changes [46]. Focusing on the HHP samples, while a* behaved similar to the blanched samples, L*, b*, and consequently C were influenced by the time/pressure interaction. Among the HHP treatments, HHP600-1 was the most affecting treatment, leading to brighter and more intense blue tones, as also confirmed by the shift of h° to values around 300 degrees. On the other hand, HHP600-5 was the least affecting HHP treatment; this may be due to better anthocyanins extraction [46], associated in our study with the high antioxidant activity of these samples.

### 3.6. Principal Component Analysis

**Figure 4:**
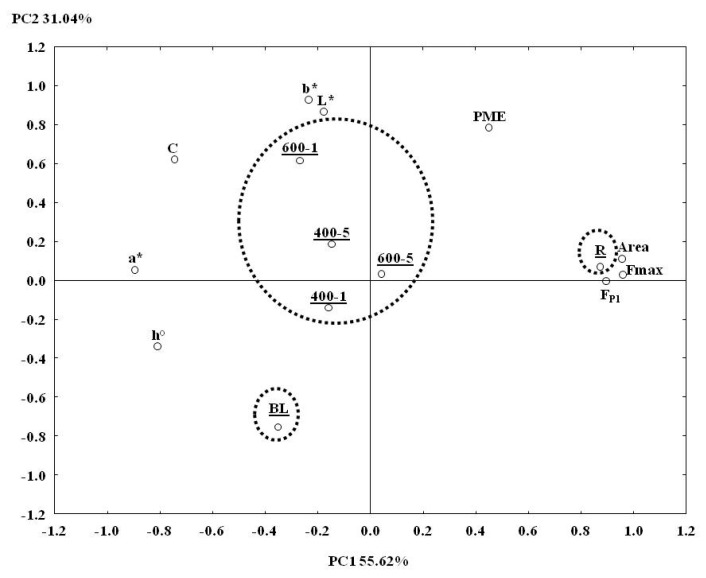
Principal Component Analysis (PCA) results obtained for the two principal components: Projection of the variables and of the cases on the factor plane (1 × 2). Abbreviations: R = raw/untreated; BL = blanched; 400-1 = HHP at 400 MPa for 1 min; 400-5 = HHP at 400 MPa for 5 min; 600-1 = HHP at 600 MPa for 1 min; 600-5 = HHP at 600 MPa for 5 min; F_P1_ = maximum force first peak; Fmax = absolute maximum force; Area = area under the force/time curve; PME = pectin methylesterase; L = lightness; a* = redness; b* = blueness; C = chroma; h° = hue angle; PC = Principal Component.

Based on the obtained results, Principal Component Analysis (PCA) (Figure 4) was performedto enable an overview of the variables that mainly influenced the final quality of blueberries under the effect of the different studied treatments. Starting from 10 variables (DPPH, PME, F_p1_, F_max_, Area, L, a*, b*, C, h°), factor analysis excluded DPPH because it showed low contribution to the total variance.

Nine variables were selected, generating a score plot in which the first two principal components (PC) explained 86.66% of the total variance. The first and more discriminating component (PC1) was related to texture and color parameters; in particular, Fp_1_, F_max_, and Area showed positive loadings on PC1, being inversely related to a*. On the second component (PC2), the color parameters L* and b* showed high positive factor loadings. The variables PME and *h*° resulted in opposite positions on the plan, having respectively positive and negative factor loadings, both on PC1 and PC2. The color parameter C showed positive loadings on PC1 and negative on PC2. Based on this distribution, the samples were grouped into three clusters: Raw, Blanched, and HHP treated samples. Raw samples were directly related with all of the texture parameters, being instead inversely related to the red component a*, indicating a more turgid structure with an opaque red color. Blanched samples were inversely related to PME and with the color parameters L and b*, having instead a positive relation with h°. The wide distance between raw and blanched samples on the factor plan confirmed that the blanched blueberries were the ones that deviated most from raw fruit in terms of organoleptic properties, however being the most effective treatment on PME inactivation. HHP treated samples clustered together in the middle zone between raw and blanched samples, indicating high pressure as a mild treatment if compared to the thermal one. Among the HHP treatments, 600-1 slightly differed from the other samples, mainly in relation to the color parameters L and b*. 

## 4. Conclusions

The application of high pressure as a fruit preservation technique has shown mild effects on the organoleptic properties of blueberries if compared to blanching, despite the more severe tissue damage. Focusing on the high-pressure treatments, an interesting effect of the pressure holding time was observed on almost all of the investigated variables. Indeed, less PME deactivation, higher texture and color retention, and better antioxidant activity were observed by increasing the treatment time for both of the tested pressures. These phenomena, probably related to an easier extraction of intracellular molecules from the broken cells, were particularly visible for 600-5, the sample subjected to the most intense high-pressure treatment. Almost no effect of the pressure level was evidenced in this study. The high PME activity, considered a defect of the high pressure treated samples, was, however, related to a better texture recovery during treatment. A shelf life study will be necessary to evaluate the evolution of these parameters over time.

## Figures and Tables

**Table 1:** Water content, antioxidant activity and residual PME activity of raw and treated blueberries ^†^.

	R	BL	400-1	400-5	600-1	600-5	P	*t*	P × *t*
DPPH (TE μmol/g_dw_)	103.4 ± 18.6 ^a^	79.1 ± 5.1 ^ab^	67.8 ± 6.5 b ^C^	79.0 ± 4.1 ^ab AB^	75.5 ± 7.5 ^b B^	84.9 ± 2.1 ^ab A^	n.s.	*	n.s.
PME (%)	100 ^a^	9.1 ± 2.14 ^c^	64.8 ± 3.7 ^b C^	95.7 ± 5.2 ^a A^	83.5 ± 9.0 ^ab B^	87.2 ± 14.2 ^a AB^	n.s.	*	*
Water content (%)	90.7 ± 1.9 ^a^	88.4 ± 0.3 ^a^	87.0 ± 0.8 ^a C^	88.0 ± 0.7 ^a B^	88.2 ± 1.1 ^a B^	89.2 ± 0.2 ^a A^	*	*	n.s

^†^ Data are expressed as means ± standard deviations of 3 samples. Means in row followed by different lowercase letters are significantly different according to the post-hoc analysis after one-way analysis of variance (ANOVA) (*p* ≤ 0.05). Means in row, of high pressure treated samples, followed by different uppercase letters are significantly different according to post-hoc comparisons after two-way ANOVA (*p* ≤ 0.05), performed considering pressure (P) and time (*t*) as independent variables. The *p* values were corrected for multiple comparisons use LSD method. Abbreviations: R = raw/untreated; BL = blanched; 400-1 = HHP at 400 MPa for 1 min; 400-5 = HHP at 400 MPa for 5 min; 600-1 = HHP at 600 MPa for 1 min; 600-5 = HHP at 600 MPa for 5 min; TE = Trolox equivalent; PME = pectin methylesterase; n.s. = not significant (*p* ≥ 0.05); * = significant (*p* ≤ 0.05).

**Table 2:** Texture parameters ^†^ for raw and treated blueberries.

Samples	F_P1_ (N)			F_max_ (N)			Area (N*s)		
R	0.30 ± 0.03 ^a^			0.32 ± 0.06 ^a^			0.79± 0.14 ^a^		
BL	0.19 ± 0.03 ^c^			0.10 ± 0.03 ^b^			0.22 ± 0.03 ^c^		
400-1	0.22 ± 0.04 ^bc AB^			0.10 ± 0.03 ^b B^			0.26 ± 0.04 ^bc B^		
400-5	0.20 ± 0.04 ^bc B^			0.10 ± 0.03 ^b B^			0.28 ± 0.06 ^bc B^		
600-1	0.20 ± 0.04 ^bc B^			0.10 ± 0.03 ^b B^			0.27 ± 0.05 ^bc B^		
600-5	0.25 ± 0.05 ^ab A^			0.14 ± 0.04 ^b A^			0.36 ± 0.08 ^b A^		
	P	*t*	P × *t*	P	*t*	P × *t*	P	*t*	P × *t*
	n.s.	n.s.	*	n.s.	n.s.	n.s.	n.s.	*	n.s.

Note: ^†^ Data are expressed as means ± standard deviations of 10 samples. Means in columns followed by different lowercase letters are significantly different according to the post-hoc analysis after one-way analysis of variance (ANOVA) (*p* ≤ 0.05). Means in columns of high-pressure treated samples followed by different uppercase letters are significantly different according to post-hoc comparisons after two-way ANOVA (*p* ≤ 0.05), performed considering pressure (P) and time (*t*) as independent variables. The *p* values were corrected for multiple comparisons use LSD method. Abbreviations: R = raw/untreated; BL = blanched; 400-1 = HHP at 400 MPa for 1 min; 400-5 = HHP at 400 MPa for 5 min; 600-1 = HHP at 600 MPa for 1 min; 600-5 = HHP at 600 MPa for 5 min; F_P1_ = maximum force first peak; F_max_ = absolute maximum force; Area = area under the force/time curve; n.s. = not significant (*p* ≥ 0.05); * = significant (*p* ≤ 0.05).

**Table 3:** Color parameters for raw and treated blueberries ^†^.

	L*			a*			b*			C			h°			ΔE		
R	31.50 ± 2.00 ^ab^			0.08 ± 0.02 ^b^			−1.66 ± 0.53 ^ab^			1.66 ± 0.53 ^c^			272.72 ± 1.94 ^c^			-		
BL	28.30 ± 3.96 ^b^			2.67 ± 0.98 ^a^			−0.41 ± 0.18 ^a^			2.72 ± 0.95 ^bc^			349.49 ± 7.50 ^a^			5.64 ± 1.10 ^a^		
400-1	30.15 ± 3.46 ^ab B^			2.52 ± 0.96 ^a A^			−1.74 ± 1.08 ^ab A^			3.19 ± 1.08 ^ab B^			326.41 ± 18.48 ^a A^			4.18 ± 1.66 ^abc AB^		
400-5	31.05 ± 2.79 ^ab B^			2.01 ± 1.07 ^a A^			−2.37 ± 1.50 ^bc A^			3.42 ± 1.02 ^ab B^			312.02 ± 24.83 ^ab AB^			3.56 ± 1.23 ^bc B^		
600-1	34.80 ± 1.69 ^a A^			2.30 ± 0.47 ^a A^			−3.61 ± 1.04 ^c B^			4.43 ± 0.53 ^a A^			304.62 ± 14.43 ^b B^			4.72 ± 1.01 ab A		
600-5	30.86 ± 1.44 ^ab B^			2.06 ± 0.80 ^a A^			−1.95 ± 0.73 ^bc A^			2.93 ± 0.73 ^b B^			317.06 ± 16.23 ^ab AB^			2.60 ± 0.73 ^c C^		
	P	*t*	P × t	P	*t*	P × *t*	P	*t*	P × *t*	P	*t*	P × *t*	P	*t*	P × *t*	P	*t*	P × *t*
	n.s.	n.s.	*	n.s.	n.s.	n.s.	n.s.	n.s.	*	n.s.	n.s.	*	n.s.	n.s.	n.s.	n.s	*	n.s.

Note: ^†^ Data are expressed as means ± standard deviations of 10 samples. Means in columns followed by different lowercase letters are significantly different according to the post-hoc analysis after one-way analysis of variance (ANOVA) (*p* ≤ 0.05). Means in columns of high-pressure treated samples followed by different uppercase letters are significantly different according to post-hoc comparisons after two-way ANOVA (*p* ≤ 0.05), and were performed considering pressure (P) and time (*t*) as independent variables. The *p* values were corrected for multiple comparisons using LSD method. Abbreviations: R = raw or untreated; BL = blanched; 400-1 = HHP at 400 MPa for 1 min; 400-5 = HHP at 400 MPa for 5 min; 600-1 = HHP at 600 MPa for 1 min; 600-5 = HHP at 600 MPa for 5 min; L = lightness; a* = redness; b* = blueness; C = chroma; *h*° = hue angle; n.s. = not significant (*p* ≥ 0.05); * = significant (*p* ≤ 0.05).
